# Platelet volume indices are associated with systolic and diastolic cardiac dysfunction, and left ventricular hypertrophy

**DOI:** 10.1186/s12872-015-0047-8

**Published:** 2015-06-16

**Authors:** Shu-ichi Fujita, Yoshihiro Takeda, Shun Kizawa, Takahide Ito, Kazushi Sakane, Toshiyuki Ikemoto, Yoshikatsu Okada, Koichi Sohmiya, Masaaki Hoshiga, Nobukazu Ishizaka

**Affiliations:** Department of Cardiology, Osaka Medical College, Takatsuki-shi Daigaku-machi 2-7, Osaka, 569-8686 Japan; Department of Central Clinical Laboratory, Osaka Medical College, Osaka, Japan

**Keywords:** Platelet function, Left ventricular hypertrophy, Cardiac function, Antithrombotic drugs

## Abstract

**Background:**

Mean platelet volume (MPV) and platelet distribution width (PDW) are indices that reflect platelet activity. We investigated the association between these platelet indices and left ventricular hypertrophy and cardiac function.

**Methods:**

We analyzed the data of 1241 patients who were admitted to the Cardiology Department.

**Results:**

Both MPV and PDW were selected as independent factors associated with left ventricular systolic and diastolic dysfunction, and left ventricular hypertrophy. The highest tertile of MPV and PDW was associated with left ventricular systolic dysfunction (left ventricular ejection fraction of <50 %) with an odds ratio of 1.53 and 2.03, respectively, when the respective lowest tertile was used as reference. The highest PDW tertile was associated with left ventricular hypertrophy with an odds ratio of 1.56 (95 % CI, 1.13–2.15) and with dysfunction with an odds ratio of 3.34 (95 % CI, 1.54–7.25).

**Conclusions:**

Indices of platelet activation (MPV and/or PDW) were independently associated positively with left ventricular hypertrophy and left ventricular systolic and diastolic dysfunction. Whether these platelet indices represent useful markers for identifying individuals at higher risk for thromboembolic disease and organ damage among cardiac patients awaits further investigation.

## Background

Activation of platelets and their subsequent aggregation play a key role in thrombus formation at the site of vascular injury and atherothrombotic events [1, 2]. Assessment of platelet activity and proper medical control are therefore mandatory for high-risk patients [3]; however, platelet aggregation after applying inducers, such as adenosine diphosphate (ADP) or 5-hydroxytryptamine, and collagen, is, in general, not measured in routine laboratory testing.

In comparison to smaller ones, larger platelets have higher thrombotic potential [4] that may be partially attributed to a higher thromboxane A2 level [5] and increased expression of glycoprotein Ib and IIb/IIIa receptors [6]. Mean platelet volume (MPV), which is the most accurate measure of platelet size, is a simple, easy to quantify, inexpensive, and widely available marker of platelet activation [7]. MPV has received substantial attention in the past few years for the purpose of risk prediction and risk stratification of various disorders, especially ischemic heart disease, in the cardiology field [4, 8–11]. Platelet distribution width (PDW), which is in general positively correlated with MPV, directly measures the variability in platelet size, and also represents a parameter of platelet activity [12]. Several previous studies have assessed PDW values among patients with acute coronary syndrome or coronary artery disease [13–15].

MPV may be increased in other cardiovascular conditions such as pulmonary arterial hypertension [16], hypertrophic cardiomyopathy [17], and decompensated heart failure [18, 19], which may explain the increase in thromboembolic events in these conditions [20], Until now, only a few studies with small sample sizes have examined the relationship between MPV and left ventricular systolic and diastolic dysfunction, and left ventricular hypertrophy [21–23]. To this end, in the current study, we analyzed the relationship between platelet indices (MPV, PDW) and left ventricular systolic and diastolic cardiac dysfunction and hypertrophy among patients admitted to the Cardiology Department.

## Methods

### Study population

The current retrospective study was approved by the Ethics Committee of Osaka Medical College. Between January 2012 and March 2014, 1241 patients who were admitted to the Cardiology Department and had provided written informed consent and for whom sufficient information regarding the data analysis for the current study including echocardiographic data was available were enrolled in the current study. Left ventricular diastolic dysfunction (LVDD) was assessed among the patients with both sinus rhythm and left ventricular ejection fraction (LVEF) of ≥50 %. Of 1241 overall study population, 821 patients were found to have both sinus rhythm and LVEF of ≥50 %. Among these patients, however, echocardiographic data that was necessary for the determination of the presence or absence of diastolic dysfunction was not available in 237 patients due to the poor echocardiographic imaging. Therefore, data from subgroup of 584 patients were used for the analysis of the relationship between platelet indices and LVDD.

### Laboratory analysis

C-reactive protein (CRP) and B-type natriuretic peptide (BNP) were measured by routine laboratory methods. The eGFR was calculated by the following Modification of Diet in Renal Disease equation for Japanese subjects: eGFR mL/min/1.73 m^2^) = 194 × (serum creatinine) ^−1.094^ × (age) ^−0.287^ (×0.739, when female) [24]. eGFR of less than 60 mL/min/1.73 m^2^ was defined as chronic kidney disease in the current study. MPV and PDW were analyzed within 2 h of venipuncture by automatic blood counter (ADVIA 2120i Hematology System; Siemens, Inc.) used for whole blood analysis, with an intra-assay coefficient of variation <1.4 % and 5.9 %, respectively.

### Echocardiography

Echocardiographic examinations were performed as described previously [25]. Briefly, left ventricular (LV) volumes were calculated using the modified Simpson method in the apical 4-chamber view. For calculation of the LV mass (LVM), we used the formula proposed by Devereux et al. [26] with modification: 0.8 × 1.04 × [(LVDd + IVST + PWT)^3^ - LVDd^3^] + 0.6. LVM index (LVMI) was calculated as the ratio of LVM to the body surface area. Left ventricular hypertrophy (LVH) was defined to be present when the LVMI was greater than 118 g/m^2^ (men) or 108 g/m^2^ (women) [27]. The LVEF was calculated by modified Simpson’s method using the apical 4-chamber view and left ventricular systolic dysfunction (LVSD) was defined to be present when LVEF was less than 50 %.

LVDD was assessed as previously described [28]. Briefly, the deceleration time of the E wave (DcT) and peak velocities of early filling (E) and atrial filling (A) were measured, and the early peak diastolic mitral annulus velocity (e’), which was the mean of that obtained at the septal and lateral mitral annulus, was measured using pulsed wave tissue Doppler. LVDD was diagnosed when any the following criteria were met; (1) E/e’ ≥ 15, (2) 15 > E/e’ ≥ 8 and BNP ≥ 200, (3) 15 > E/e’ ≥ 8, E/A < 0.5, and DcT ≥ 280 msec, (4) 15 > E/e’ ≥ 8, E/A < 0.5 and presence of LVH.

### Statistical analysis

Baseline characteristics were assessed with standard descriptive statistics. Data were expressed as either mean ± standard deviation or median and interquartile range. A Pearson’s correlation test was used to assess the correlation between two variables. For multivariate analysis, multivariate linear regression and multivariate logistic regression analyses were used. Data analysis was performed by SPSS statistics version 22.0 (IBM, Armonk, NY). A value of *P* < 0.05 was taken to be statistically significant.

## Results

### Patient characteristics

**Fig. 1:**
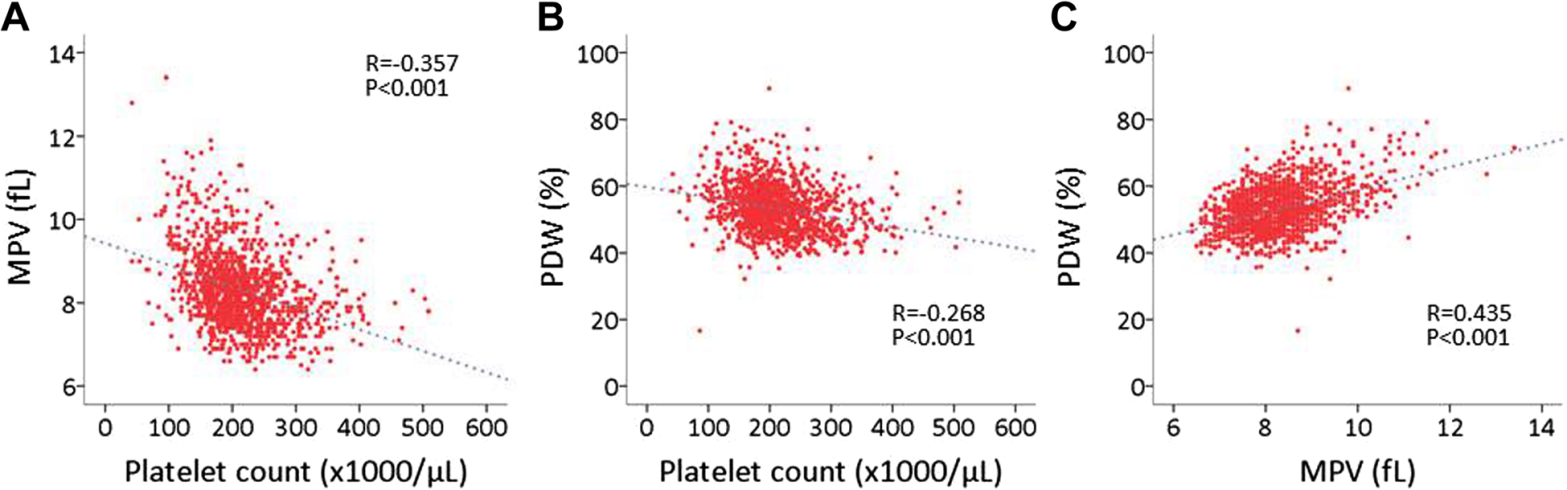
Correlation between platelet indices. **a** Correlation between platelet count and mean platelet volume (MPV). **b** Correlation between platelet count and platelet distribution width (PDW). **c** Correlation between MPV and PDW

Among the 1241 patients enrolled, 910 were male (73 %) and 1008 (81 %) had sinus rhythm (Table 1). Only 53 (4 %) patients were undergoing chronic hemodialysis. Platelet count was significantly negatively associated with MPV and PDW, and MPV showed a significant positive correlation with PDW (Fig. 1). Echocardiography showed that 263 (21 %) patients had LVSD and 448 (36 %) had LVH.

**Table 1:** Clinical characteristics of the study patients

Variables	Women (*n* = 331)	Men (*n* = 910)
Age, years	71.1 ± 10.9	68.3 ± 10.8
Body mass index, kg/m^2^	22.9 ± 4.1	23.7 ± 3.5
Chronic hemodialysis, n (%)	8 (2.4)	45 (4.9)
Ever smoker	55 (16.6)	721 (79.2)
Cardiac rhythm		
Sinus rhythm, n (%)	258 (77.9)	750 (82.4)
Atrial fibrillation, n (%)	45 (13.6)	107 (11.8)
Pacemaker, n (%)	17 (5.1)	38 (4.2)
Others, n (%)	11 (3.3)	15 (1.6)
Cardiovascular disease		
Ischemic heart disease, n (%)	173 (52.3)	681 (74.8)
Arrhythmic disease, n (%)	35 (10.6)	70 (7.7)
Cardiomyopathy, n (%)	113 (34.1)	217 (23.8)
Peripheral artery disease, n (%)	15 (4.5)	82 (9.0)
Valvular heart disease, n (%)	34 (10.3)	71 (7.8)
Medication		
ACE inhibitors/ARB, n (%)	150 (45.3)	524 (57.6)
Beta blockers, n (%)	123 (37.2)	372 (40.9)
Calcium channel blockers, n (%)	162 (48.9)	389 (42.7)
Loop diuretics, n (%)	102 (30.8)	202 (22.2)
Thiazide diuretics, n (%)	25 (7.6)	38 (4.2)
Aldosterone antagonist, n (%)	35 (10.6)	79 (8.7)
Aspirin, n (%)	166 (50.2)	658 (72.3)
Clopidogrel, n (%)	88 (26.6)	393 (43.2)
Any antiplatelet drug, n (%)	176 (53.2)	689 (75.7)
Warfarin, n (%)	78 (23.6)	202 (22.2)
NOAC, n (%)	40 (12.1)	79 (8.7)
Any anticoagulants, n (%)	118 (35.6)	281 (30.9)
Laboratory data		
White blood cell count, x10^3^/μL	5.67 (4.60–6.86)	6.07 (4.98–7.27)
Hemoglobin, g/dL	12.4 (11.2–13.5)	13.6 (12.3–14.8)
Platelet count, x10^3^/μL	212 (172–259)	201 (171–239)
Mean platelet volume, fL	8.1 (7.7–8.7)	8.2 (7.7–8.8)
Platelet distribution width, %	52.0 (47.3–56.2)	53.1 (48.3–58.0)
Serum creatinine, mg/dL	0.72 (0.62–0.93)	0.92 (0.80–1.13)
eGFR, mL/min/1.73 m^2^	60.4 (45.9–71.8)	47 (37.3–55.9)
Echocardiographic data		
LV diastolic dimension, cm	4.6 (4.2–5.1)	4.9 (4.6–5.5)
LV systolic dimension, cm	2.9 (2.5–3.5)	3.3 (2.9–3.9)
LV ejection fraction, %	61 (54–68)	59 (51.0–65)
LV mass index, g/m^2^	99 (83–123)	106 (89.9–128)

ACE, angiotensin converting enzyme; ARB, angiotensin receptor blockers; NOAC, non-warfarin novel oral anticoagulants. For the data of serum creatinine and eGFR, patients on chronic hemodialysis (*n* = 53) were excluded from the analysis

### Relationship between antithrombotic drug usage and platelet indices

**Fig. 2:**
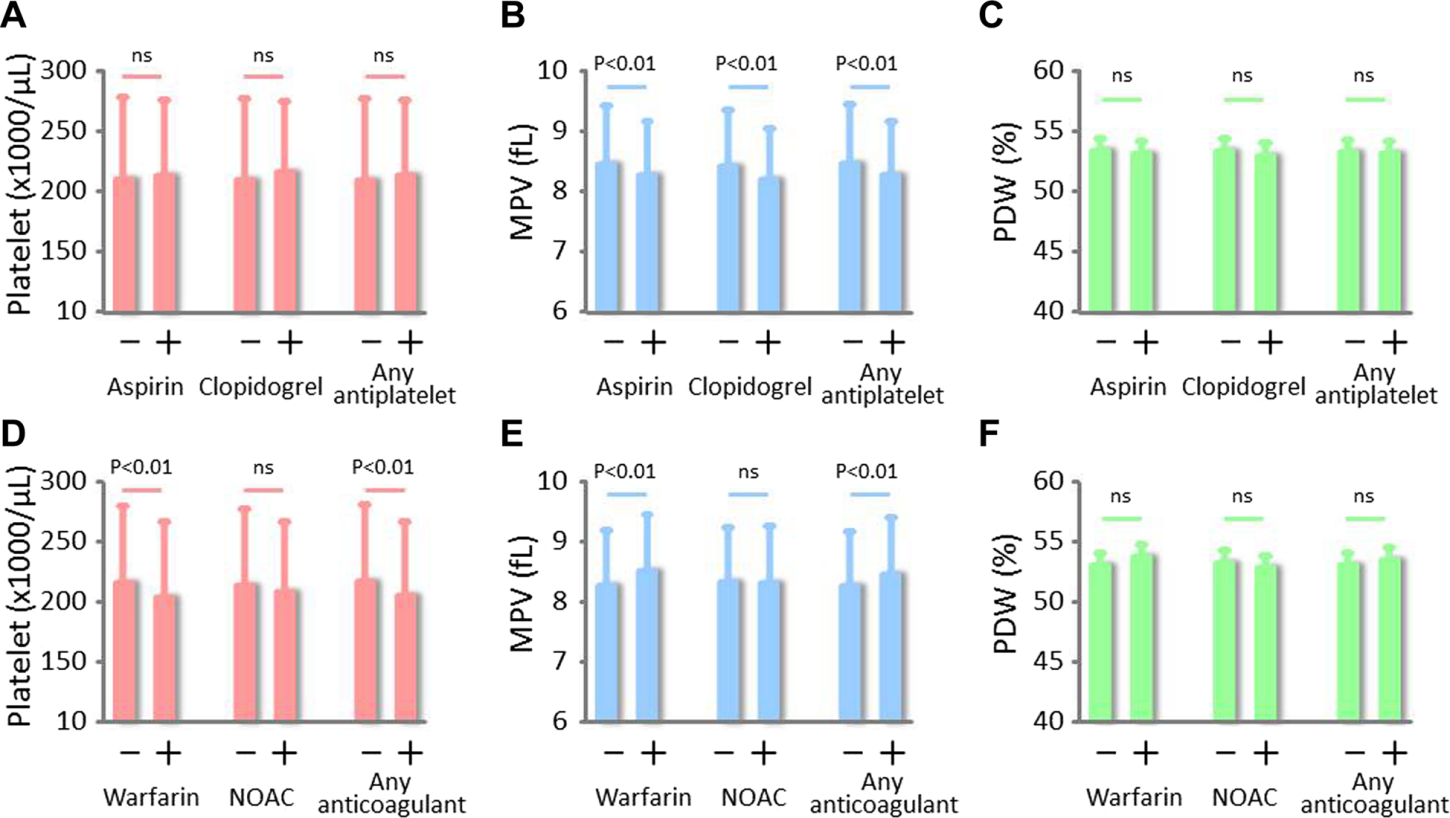
Platelet indices according to antiplatelet or anticoagulative medication. Shown are platelet count (**a**), platelet volume (MPV) (**b**), and platelet distribution width (PDW) (**c**) according to antiplatelet drug use, and platelet count (**d**), MPV (**e**), and PDW (**f**) according to anticoagulant medication use

About one third of the study patients were taking anticoagulant medication and more than half were taking aspirin and/or other antiplatelet drugs. Some platelet indices differed between the anti-thrombotic drug users and the non-users (Fig. 2); patients taking aspirin or clopidogrel had significantly lower MPV as compared with non-users (Fig. 2b). In addition, patients taking warfarin had a lower platelet count and higher MPV as compared with non-users (Fig. 2d, e), although these values did not significantly differ according to the use and non-use of non-warfarin novel oral anticoagulants (NOAC).

It was found that the MPV value in patients with atrial fibrillation (8.59 ± 0.91 fL) was significantly higher than that in patients with sinus rhythm (8.25 ± 0.87 fL, *P* < 0.001). When the comparison was limited to patients with sinus rhythm, the MPV values in patients with warfarin usage and that in patients without warfarin usage did not differ significantly (8.24 ± 0.87 fL versus 8.34 ± 0.87 fL, respectively, *P* = 0.202). In addition, MPV did not differ significantly between warfarin users and non-users among patients with sinus rhythm who were taking at least one anti-platelet medication (8.33 ± 0.86 fL [*n* = 92] versus 8.21 ± 0.87 fL [*n* = 669], *P* = 0.203) or among patients with sinus rhythm who were not taking any anti-platelet medication (8.35 ± 0.91 fL [*n* = 53] versus 8.35 ± 0.87 fL [*n* = 194], *P* = 0.959).

### Relationship between platelet indices and left ventricular systolic function and hypertrophy

**Fig. 3:**
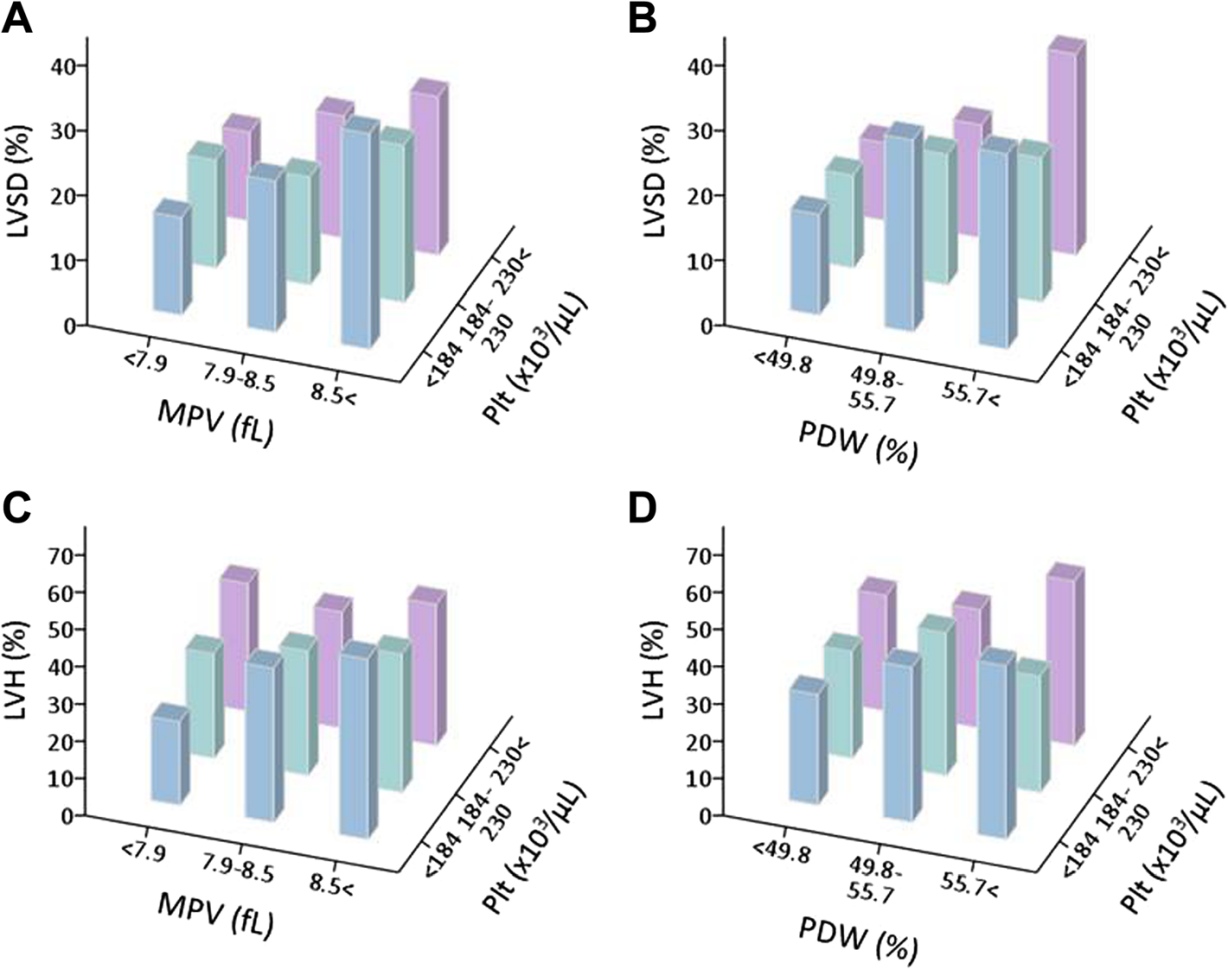
Prevalence of left ventricular systolic dysfunction (LVSD) and left ventricular hypertrophy (LVH) according to platelet indices. Shown is the prevalence of LVSD (**a**, **b**) and LVH (**c**, **d**) according to platelet count and mean platelet volume (MPV) tertiles (**a**, **c**), and platelet count and platelet distribution width (PDW) tertiles (**b**, **d**)

When the data were assessed in tertiles, the prevalence of LVSD seemed to increase according to the MPV (Fig. 3a) or PDW (Fig. 3b) tertile, but not the platelet count tertile. This tendency seemed to be less apparent for the prevalence of LVH (Fig. 3c, d). In univariate linear regression analysis, platelet count, MPV, and PDW were each significantly associated with both LVEF and LVMI (Table 2). When stepwise multivariate analysis was performed by entering all of the variables that used in univariate analysis, MPV and PDW, but not platelet count, were found to have a significant association with LVEF and LVMI.

**Table 2:** Linear regression analysis of factors associated with LVEF and LVMI

	Univariate		Multivariate (stepwise)	
	Std β	*P* value	Std β	*P* value
Dependent variable: LVEF				
Sex (male = 1)	−0.10	<0.001	-	
Age	0.07	0.011	0.14	<0.001
Systolic blood pressure	0.07	0.009	0.06	0.021
Chronic kidney disease	−0.12	<0.001	−0.12	<0.001
White blood cell count	−0.09	0.001	−0.06	0.034
Hemoglobin	0.06	0.047	0.08	0.007
Platelet	0.08	0.004	-	
MPV	−0.19	<0.001	−0.12	<0.001
PDW	−0.18	<0.001	−0.13	<0.001
Dependent variable: LVMI				
Sex (male = 1)	0.05	0.078	-	
Age	0.09	0.002	-	
Systolic blood pressure	0.09	0.002	0.09	0.001
Chronic kidney disease	0.15	<0.001	0.12	<0.001
White blood cell count	0.07	0.014	-	
Hemoglobin	−0.17	<0.001	−0.15	<0.001
Platelet count	−0.09	0.001	-	
MPV	0.15	<0.001	0.08	0.015
PDW	0.10	<0.001	0.07	0.016

### Multivariate logistic regression analysis of the relationship with left ventricular systolic dysfunction and hypertrophy

Next, we performed multivariate logistic regression analyses using LVSD or LVH as a dependent variable (Tables 3 and 4). In this analysis, use of antiplatelet drugs and use of warfarin were entered as independent variables. Warfarin use was found to be, respectively, significantly and borderline significantly positively associated with LVSD and LVH. In model 1, where platelet indices were entered on a per 1 standard deviation (SD) basis, PDW was, respectively, significantly and borderline significantly associated with LVSD and LVH. In this model, the association between MPV and LVH was not significant; however, in an analysis adjusted for sex, age, anti-platelet drug use, and warfarin use, MPV was significantly associated with LVSD with an odds ratio of 1.26 (95 % CI 1.12–1.42, per 1 SD, *P* < 0.001).

**Table 3:** Multivariate logistic regression analysis of factors associated with LVSD

Independent variables	Odds ratio (95 % CI)	*P* value	Odds ratio (95 % CI)	*P* value
	model 1		model 2	
Sex (male = 1)	1.08 (0.74–1.58)	0.700	1.07 (0.73–1.56)	0.743
Age, per 1SD	0.83 (0.71–0.97)	0.018	0.83 (0.71–0.97)	0.017
Systolic blood pressure, per 1SD	0.87 (0.75–1.01)	0.059	0.87 (0.75–1.01)	0.062
Chronic kidney disease	1.56 (1.03–2.36)	0.036	1.60 (1.05–2.43)	0.027
Any antiplatelet drugs	1.00 (0.72–1.38)	0.997	0.98 (0.71–1.35)	0.888
Warfarin	2.18 (1.58–2.99)	<0.001	2.18 (1.58–2.99)	<0.001
White blood cell count, per 1SD	1.19 (1.03–1.37)	0.016	1.18 (1.03–1.36)	0.017
Hemoglobin, per 1SD	0.78 (0.67–0.90)	0.001	0.79 (0.68–0.91)	0.001
Platelet count, per 1SD	0.90 (0.76–1.06)	0.206		
MPV, per 1SD	1.15 (0.98–1.35)	0.096		
PDW, per 1SD	1.25 (1.06–1.46)	0.007		
Middle platelet tertile			0.80 (0.56–1.14)	0.218
Highest platelet tertile			0.83 (0.56–1.23)	0.346
Middle MPV tertile			1.18 (0.80–1.73)	0.412
Highest MPV tertile			1.53 (1.04–2.27)	0.033
Middle PDW tertile			1.80 (1.22–2.64)	0.003
Highest PDW tertile			2.03 (1.37–3.02)	<0.001

In model 2, platelet indices used in model 1 were used replaced by tertile of these variables, and the odds ratio of the middle and the highest tertile was calculated using the corresponding lowest tertile

**Table 4:** Multivariate logistic regression analysis of factors associated with LVH

Independent variables	Odds ratio (95 % CI)	*P* value	Odds ratio (95 % CI)	*P* value
	model 1		model 2	
Sex (male = 1)	0.87 (0.63–1.19)	0.386	0.86 (0.62–1.18)	0.356
Age, per 1SD	0.93 (0.81–1.06)	0.264	0.93 (0.81–1.06)	0.276
Systolic blood pressure, per 1SD	1.22 (1.08–1.37)	0.002	1.22 (1.08–1.38)	0.001
Chronic kidney disease	1.69 (1.20–2.36)	0.002	1.75 (1.25–2.46)	0.001
Any antiplatelet drugs	0.80 (0.61–1.05)	0.103	0.79 (0.60–1.04)	0.092
Warfarin	1.30 (0.97–1.74)	0.075	1.30 (0.97–1.74)	0.074
White blood cell count, per 1SD	1.12 (0.99–1.27)	0.074	1.11 (0.98–1.26)	0.101
Hemoglobin, per 1SD	0.72 (0.63–0.82)	<0.001	0.72 (0.63–0.82)	<0.001
Platelet count, per 1SD	0.93 (0.81–1.07)	0.303		
MPV, per 1SD	1.10 (0.96–1.27)	0.171		
PDW, per 1SD	1.12 (0.98–1.29)	0.095		
Middle platelet tertile			0.80 (0.59–1.09)	0.160
Highest platelet tertile			0.94 (0.67–1.31)	0.697
Middle MPV tertile			1.15 (0.84–1.56)	0.388
Highest MPV tertile			1.28 (0.92–1.77)	0.139
Middle PDW tertile			1.39 (1.02–1.88)	0.035
Highest PDW tertile			1.56 (1.13–2.15)	0.007

In model 2, platelet indices used in model 1 were used replaced by tertile of these variables, and the odds ratio of the middle and the highest tertile was calculated using the corresponding lowest tertile

In model 2, where platelet indices were entered as tertile basis, the highest tertile of MPV and PDW was associated with LVSD with an odds ratio of 1.53 and 2.03, respectively, compared with the respective lowest tertile (Table 3).

### Relationship between platelet indices and left ventricular diastolic dysfunction

Next, the relationship between platelet indices and LVDD was investigated. Among 584 patients for whom presence or absence of diastolic dysfunction was assessed, 71 patients (12.2 %) were found to have LVDD. In model 1 where platelet indices were entered on a per 1 SD basis, platelet count and PDW, respectively, were found to be significantly negatively and positively associated with LVDD (Table 5). In this model, the association between MPV and LVDD was not significant; however, in an analysis adjusted for sex, age, anti-platelet drug use, and warfarin use, MPV was significantly associated with LVDD with an odds ratio of 1.34 (95 % CI, 1.02–1.76, per 1 SD, *P* = 0.036). In model 2, where platelet indices were entered on a tertile basis, the highest tertile of PDW was associated with LVDD with an odds ratio of 3.34 compared with the lowest tertile (Table 5).

**Table 5:** Multivariate logistic regression analysis of factors associated with LVDD

Independent variables	Odds ratio (95 % CI)	*P* value	Odds ratio (95 % CI)	*P* value
	model 1		model 2	
Sex (male = 1)	0.42 (0.21–0.84)	0.014	0.37 (0.18–0.75)	0.006
Age, per 1SD	1.34 (0.95–1.89)	0.095	1.30 (0.92–1.83)	0.139
Systolic blood pressure, per 1SD	1.17 (0.89–1.56)	0.263	1.20 (0.91–1.60)	0.200
Chronic kidney disease	2.43 (1.12–5.30)	0.025	2.78 (1.24–6.24)	0.013
Any antiplatelet drugs	0.59 (0.30–1.15)	0.123	0.65 (0.33–1.29)	0.220
Warfarin	1.11 (0.47–2.61)	0.810	1.11 (0.47–2.62)	0.811
White blood cell count, per 1SD	1.54 (1.14–2.07)	0.005	1.52 (1.13–2.03)	0.005
Hemoglobin, per 1SD	0.50 (0.37–0.66)	<0.001	0.51 (0.38–0.67)	<0.001
Platelet count, per 1SD	0.62 (0.46–0.86)	0.003		
MPV, per 1SD	0.92 (0.66–1.29)	0.641		
PDW, per 1SD	1.41 (1.03–1.92)	0.031		
Middle platelet tertile			0.42 (0.21–0.82)	0.011
Highest platelet tertile			0.32 (0.15–0.67)	0.003
Middle MPV tertile			1.00 (0.49–2.04)	0.991
Highest MPV tertile			0.88 (0.42–1.86)	0.738
Middle PDW tertile			1.91 (0.90–4.05)	0.092
Highest PDW tertile			3.34 (1.54–7.25)	0.002

In model 2, platelet indices used in model 1 were used replaced by tertile of these variables, and the odds ratio of the middle and the highest tertile was calculated using the corresponding lowest tertile

## Discussion

In the current study, we analyzed platelet indices and cardiac hypertrophy and left ventricular systolic and diastolic function among cardiac patients. In a univariate analysis, platelet count, MPV, and PDW were each correlated with LVEF and LVMI; however, stepwise multivariate regression analysis showed that MPV and PDW, but not platelet count, were independently associated with LVEF and LVMI. By multivariate logistic regression analysis, the highest tertile of MPV and PDW was associated with left ventricular systolic dysfunction (LVEF < 50 %) with an odds ratio of 1.53 and 2.03, respectively, as compared with the respective lowest tertile (Table 3). Platelet count and PDW were found to be independently associated with LVH and with LVSD. MPV was associated with both LVH and LVSD after adjusting for sex, age, anti-platelet drug usage, and warfarin usage; however, after further adjustment for covariates including platelet count and PDW, these associations lost statistical significance, which was explained, at least in part, by the inter-relationship among platelet count, MPV, and PDW (Fig. 1).

Some studies previously showed the relationship between MPV and cardiac function/hypertrophy [19, 21–23]. In the current study, more than 1200 patients were studied for the association between platelet indices and cardiac systolic dysfunction/hypertrophy, and more than 580 patients were studied for the association between platelet indices and cardiac diastolic dysfunction. We also carefully assessed whether the antithrombotic medication use affected the observed relation between platelet indices and cardiac parameters. In addition, there have been no studies examining the relationship between platelet indices and heart failure in Japanese population.

Among the current study patients, approximately one-third and more than half, respectively, were taking anticoagulant medication and antiplatelet drugs. MPV values were lower among patients who were taking either aspirin or clopidogrel as compared with those who were not (Fig. 2b). Some studies have demonstrated that aspirin [29, 30] or dual antiplatelet therapy [31] may not significantly affect MPV, and one study showed a paradoxical increase in MPV after antiplatelet therapy was started [32], which might be related to a lack of patients response to clopidogrel [33]. The reason why antiplatelet drugs reduced MPV in the current study is unclear. Whether lower MPV values predict cardiovascular risk or restenosis after percutaneous coronary intervention among patients taking antiplatelet drugs [34] awaits future investigation.

Warfarin, but not NOAC, usage was found to be associated with the increased MPV value (Fig. 2e). Arik previously reported that MPV was not significantly altered by warfarin when it effectively prolonged international normalized ratio among patients with non-valvular atrial fibrillation [35]. It has been reported that MPV is higher in patients with atrial fibrillation than in those with sinus rhythm [36, 37]. Because the association between warfarin use and MPV lost statistical significance, irrespective of antiplatelet drug use, when limited to the population with sinus rhythm in the current study, the association observed between warfarin and MPV may be attributed to the presence of atrial fibrillation. We also showed that NOAC did not affect platelet factors. In several previous studies, a relationship between MPV and left ventricular systolic function and hypertrophy has been reported: for example, MPV has been found to have a positive [22, 38] or no [39–41] association with LVH and a negative association with LVEF [21, 42]. On the other hand, most studies did not take PDW or antithrombotic drug use into account.

LVDD may increase the risk of stroke [43, 44]. Although previous studies demonstrated the platelet activation in patients with heart failure [20, 45], a relationship between platelet factors in patients with LVSD seems not to have been specifically investigated. Our study found that platelet count and PDW, respectively, were negatively and positively associated with LVDD among the subgroup of patients who had sinus rhythm and preserved left ventricular systolic function (Table 5). MPV was not independently associated with LVDD after full adjustment including PDW, which might be related to the fact that PDW is a more specific marker of platelet activation [46]. Notably, however, MPV was associated with LVDD with an odds ratio of 1.34 (95 % CI 1.02–1.76) in a model adjusted for sex, age, and use of anti-platelet drugs and warfarin. Whether PDW and MPV are useful parameters for discriminating patients with LVDD and those at higher risk for future thromboembolic risk and target organ damage [38] needs to be investigated in future studies.

There are several limitations to the current study. First, we did not directly assess the platelet aggregation induced by ADP or other agonists. Second, because of the cross-sectional nature of the study, we did not have data regarding future thromboembolic risk among patients with high MPV or PDW value. Third, whether MPV and PDW are useful predictors of future thromboembolic risk and targets for pharmacological intervention should be investigated in future prospective studies. Fourth, as we did not directly measure the platelet function, some conclusions may be based on the speculative discussion. On the other hand, as MPV and PDW are easily acquired parameters, we may verify whether measurement of these parameters is of use for prediction of cardiovascular events in future studies.

## Conclusion

In conclusion, we herein demonstrated that usage of antiplatelet drug and warfarin was related to MPV, and that the platelet indices MPV and PDW, but not platelet count, were independently associated with left ventricular systolic function and mass index. Among patients with preserved systolic function and sinus rhythm, the highest PDW tertile was associated with LVDD with an odds ratio of 1.56 (95 % CI, 1.13–2.15) when compared with the lowest PDW tertile after multivariate adjustment. Whether these platelet indices represent useful markers for discriminating those at higher risk for thromboembolic disease and organ damage among cardiac patients awaits further investigation.
